# Cell mediated remodeling of stiffness matched collagen and fibrin scaffolds

**DOI:** 10.1038/s41598-022-14953-w

**Published:** 2022-07-11

**Authors:** Alicja Jagiełło, Ulysses Castillo, Elliot Botvinick

**Affiliations:** 1 Department of Biomedical Engineering, University of California, Irvine, CA 92697-2715 USA; 2 Beckman Laser Institute and Medical Clinic, University of California, Irvine, CA 92612 USA; 3 Department of Surgery, University of California Irvine, 333 City Boulevard, Suite 700, Orange, CA 92868 USA; 4 The Edwards Lifesciences Foundation Cardiovascular Innovation and Research Center, University of California, Irvine, CA 92697-2730 USA

**Keywords:** Optical manipulation and tweezers, Biomaterials - cells, Biomedical engineering

## Abstract

Cells are known to continuously remodel their local extracellular matrix (ECM) and in a reciprocal way, they can also respond to mechanical and biochemical properties of their fibrous environment. In this study, we measured how stiffness around dermal fibroblasts (DFs) and human fibrosarcoma HT1080 cells differs with concentration of rat tail type 1 collagen (T1C) and type of ECM. Peri-cellular stiffness was probed in four directions using multi-axes optical tweezers active microrheology (AMR). First, we found that neither cell type significantly altered local stiffness landscape at different concentrations of T1C. Next, rat tail T1C, bovine skin T1C and fibrin cell-free hydrogels were polymerized at concentrations formulated to match median stiffness value. Each of these hydrogels exhibited distinct fiber architecture. Stiffness landscape and fibronectin secretion, but not nuclear/cytoplasmic YAP ratio differed with ECM type. Further, cell response to Y27632 or BB94 treatments, inhibiting cell contractility and activity of matrix metalloproteinases, respectively, was also dependent on ECM type. Given differential effect of tested ECMs on peri-cellular stiffness landscape, treatment effect and cell properties, this study underscores the need for peri-cellular and not bulk stiffness measurements in studies on cellular mechanotransduction.

## Introduction

Extracellular matrix (ECM) provides physical and biochemical support and controls homeostasis, function and development of majority of eukaryotic cells in the human body. While natural ECM is mostly comprised of type I collagen (T1C), even small changes in composition or biochemical and mechanical properties of the ECM can alter cell development and behavior^1–4^. For instance, bulk stiffness of the ECM was found to regulate cell morphology, rate of migration, stem cell differentiation and cancer cell invasiveness^5–8^. In a reciprocal way, ECM is also constantly remodeled by the cells^2–4^. Past research by our group has shown that 24 h after hydrogel polymerization, cells alter local and peri-cellular stiffness by a few orders of magnitude, even on a single cell level^9^. Further, cells can promote distinct stiffness anisotropies which vary with cell line, T1C concentration and biochemical treatments^10^. Nonetheless, a comprehensive understanding of bi-directional relationship between cell behavior and ECM stiffness is still lacking. Of note, most existing studies on cell mechanotransduction in three-dimensional (3D) tissue culture fail to correlate local ECM properties and cell protein expression. This deficiency can be explained by a lack of tools for making such measurements. It is widely known that cells sense their peri-cellular ECM stiffness via complexes associated with trans-membrane mechanosensory receptors that mediate attachment to ECM fibers^11,12^. Externally applied loads, or cell-generated contractile forces are then converted into biochemical signals in response to deformation of these attachments^13,14^. One such potent biochemical signal is translocation of the transcriptional factor Yes-associated protein (YAP) from the cytoplasm into the nucleus. This translocation is part of the Hippo pathway, involved in regulating gene expression in processes related to cell proliferation, differentiation and cancer progression^13,14^. In one aspect of this study, we seek correlations between local ECM stiffness measured by optical tweezers and YAP translocation within three types of naturally derived ECMs formulated to vary protein concentration and initial or cell-free stiffness.

In this study, we assessed the effect of hydrogel concentration and ECM type on stiffness around two distinct types of cells—normal human dermal fibroblasts (DFs) and human fibrosarcoma HT1080 cells. Our laboratory uses multi-axes optical tweezers active microrheology (AMR) to measure stiffness around cells along four distinct axes. In the first set of experiments, cells were cultured inside rat tail T1C hydrogels prepared at 4 different concentrations (1.0, 1.5, 2.0 and 3.0 mg/ml). AMR measurements were conducted 48 h after sample preparation to investigate if local stiffness levels established by the cells are influenced by the initial concentration of hydrogels or whether cells have a stiffness setpoint and remodel their local environment to promote stiffness levels they intrinsically prefer, as previously suggested by our group^10^.

In the second set of experiments, cells were subjected to three different types of ECM—rat tail T1C, bovine skin T1C and fibrin. While rat tail T1C is most frequently used in in vitro research^15,16^, bovine skin T1C is more commonly utilized in therapeutic applications, due to its minimal antigenicity^16,17^. Conversely, less durable fibrin forms temporary and provisional matrix during wound healing processes^18,19^. At much higher concentration, fibrin sealant is used as a tissue adhesive approved for laboratory and clinical use^20–22^. Hydrogel mechanics, fiber architecture and porosity are known to vary between different ECMs^15,16,19,23,24^, yet studies comparing properties of hydrogels prepared at similar concentrations often fail to account for difference in stiffness sensed by the cells. Consequently, in this study, concentrations of rat tail T1C, bovine skin T1C and fibrin were chosen to ensure similar initial stiffness of hydrogels. The effect of ECM on cell behavior was further investigated by analyzing the impact of two small molecules known to inhibit cell contractility or matrix digestion. The first molecule is the Rho-kinase inhibitor Y27632 known to reduce cell contractility and migration of fibroblasts^25–27^ and HT1080s^28–31^. The second molecule is the broad-spectrum zinc metalloproteinase (MMP) inhibitor batimastat (also known as BB94), previously shown to prevent MMP secretion^32–34^ and collagenolytic activity of DFs and HT1080s^34^. Past studies by our group^9^ further found that both Y27632 and BB94 promote decrease in stiffness around the DFs, but the effect of type of ECM on cell response to treatments has not been previously investigated.

Results reported in this study demonstrate that peri-cellular stiffness of DFs and HT1080s varies depending on type of ECM, hydrogel concentration and tested treatment. Interestingly, cell properties including nuclear/cytoplasmic YAP ratio and proportion of secreted fibronectin do not correlate with peri-cellular stiffness levels in our experiments. Cell environment is shown to affect cellular response to treatments, underscoring the need for peri-cellular and not bulk stiffness measurements in studies on cellular mechanotransduction.

## Results

### Fibroblast response to type 1 collagen ECM of increasing concentration

**Figure 1:**
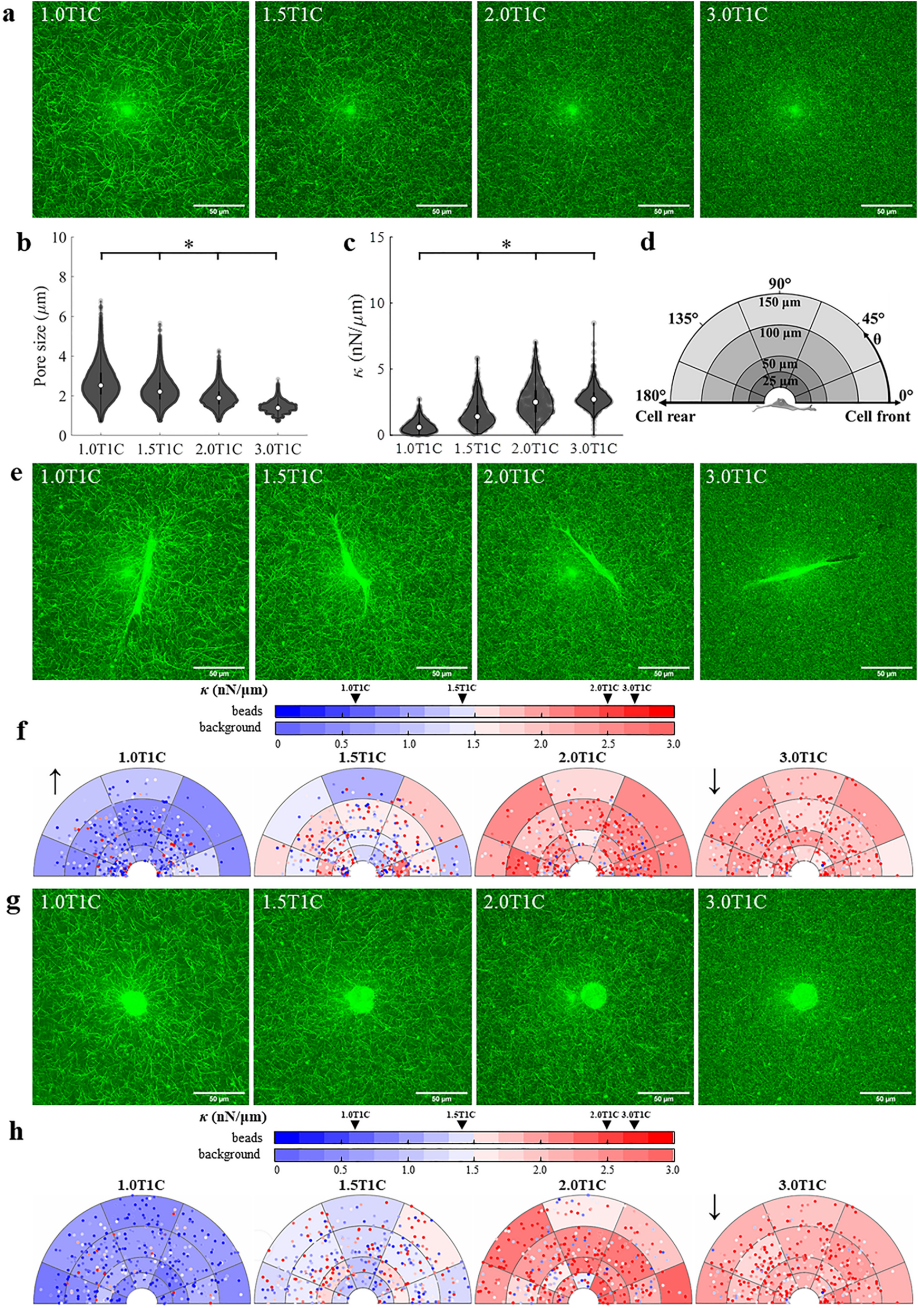
The effect of rat tail T1C concentration on stiffness distribution around DF and HT1080 cells. (**a**) Reflection confocal microscopy (RCM) images of cell-free hydrogels prepared at 4 concentrations (1.0, 1.5, 2.0, 3.0 mg/ml). (**b**) Pore size and (**c**) stiffness (*κ*) distribution of cell-free hydrogels (*n*_sample_ = 3 per concentration, *n*_beads_ > 40 per sample). Median values are denoted by white markers. (**d**) Graphical representation of the coordinate system used to discretize the ECM region around a cell. (**e**) RCM images of DFs. (**f**) Distribution of stiffness around DFs probed along the long axis of the cell (*n*_sample_ = 5, *n*_cells_ = 10 per concentration, *n*_beads_ > 40 per cell). (**g**) RCM images of HT1080s. (**h**) Distribution of stiffness around HT1080s probed along the horizontal direction of the image (*n*_sample_ = 5, *n*_cells_ = 10 per concentration, *n*_beads_ > 40 per cell). Background color is shaded according to the median *κ* value in each bin (background color bar). Each data point is a single probed bead, color-coded for *κ* (beads color bar). Black arrows indicate a significant difference in *κ* (*p* < 0.05) as compared to cell-free hydrogels of matched concentration. Arrow direction indicates increased or decreased stiffness. *p < 0.05.

Past studies by our group observed comparable stiffness levels around DFs cultured for 24 h in rat tail T1C prepared at 1.0 mg/ml and 1.5 mg/ml^10^. In order to more thoroughly explore the effect of initial collagen concentration on peri-cellular stiffness, DFs were cultured for 48 h inside rat tail T1C hydrogels polymerized at 4 different concentrations—1.0 mg/ml (1.0T1C), 1.5 mg/ml (1.5T1C), 2.0 mg/ml (2.0T1C) and 3.0 mg/ml (3.0T1C, Fig. 1a; bright spot located at the center of each image is an imaging artifact common to reflection confocal microscopy). Prior to cell experiments, properties of cell-free hydrogels were assessed. Median pore size decreased with concentration from 2.52 μm in 1.0T1C hydrogels to 1.39 μm in 3.0T1C hydrogels (Fig. 1b). Stiffness (*κ*) of hydrogels was probed by oscillating at least 40 microbeads per hydrogel at 50 Hz along 4 different axes—at 0°, 45°, 90°, 135° with respect to the horizontal axis of the image (*n*_*sample*_ = 3). Just prior to each ECM measurement, AMR was first conducted in water to validate no bias towards any of the probed axes. As expected, *κ* of cell-free hydrogels increased with concentration (Fig. 1c) and *κ* was found to be isotropic at each concentration (Fig. S1). A negative correlation was detected between *κ* and pore size (ρ = − 0.94; *p* = 0.06) indicating that stiffness increased with decreasing pore size.

Next, *κ* was measured around DFs, which were found to have similar elongated morphology when cultured at all tested concentrations of rat tail T1C (Fig. 1e). In order to minimize the effect of 3D spreading and remodeling, cells chosen for AMR experiments were predominantly in focus in the *XY* plane. Stiffness was probed at 0°, 45°, 90°, 135° with respect to the long axis of the cell and graphed as illustrated in Fig. 1d. For each probed bead, two coordinates were determined—(a) the shortest distance between the bead and cell profile and (b) angular position $\theta$ relative to the long axis of the cell in the counterclockwise direction, with origin at the cell centroid. Thus, each bead was placed within one of the twenty annular bins. Each bin is shaded according to the median *κ* value based on all beads analyzed in a particular bin. Each point corresponds to a probed bead and is color-codded for *κ*. Under the assumption of symmetry, the coordinate system was folded upon itself along the long axis of the cell. Spatial distribution of *κ* probed along the long axis of DFs is shown in Fig. 1f, while *κ* probed in other directions is represented in Fig. S2a–c.

When analyzing all beads probed around DFs along all 4 axes (Fig. S2d), *κ* around cells was found to be higher than cell-free stiffness in 1.0T1C hydrogels (*p* < 0.01), but not different from cell-free stiffness in 1.5T1C (*p* = 0.96) and 2.0T1C (*p* = 0.63) hydrogels. For 3.0T1C hydrogels, stiffness around DFs was actually lower than cell-free stiffness (*p* < 0.01) at that concentration and comparable with *κ* around DFs embedded inside 2.0T1C hydrogels (*p* = 0.88).

Multivariate exponential regression (MER) was used to determine significant predictors of *κ* from multiple independent parameters (Fig. S2e). Analyzed parameters included the continuous variables of shortest distance between the bead and cell profile and angular position $\theta$, as well as the discrete variables of T1C concentration and axis of bead oscillation. Discrete variables were simply encoded with the reference to 1.0T1C concentration and oscillation along the long axis of the cell^35^. MER indicated that T1C concentration was a dominant predictor. *κ* increased with T1C concentration, but decreased with $\theta$ position. Surprisingly, distance away from the cell (up to 150 μm) and axes of bead oscillation were not found to be significant predictors of *κ* probed 48 h after sample preparation. This finding is in contrast to our previously published data collected 24 h after sample preparation^10^. Analysis of the peri-cellular region (< 25 μm, inner annulus) similarly found that *κ* was isotropic in 1.0T1C (*p* = 0.93), 1.5T1C (*p* = 0.58), 2.0T1C (*p* = 0.75) and 3.0T1C (*p* = 0.72) hydrogels. The parameters analyzed here were only able to explain 31.5% of the variance in *κ*, indicating that factors outside our consideration play an important role in predicting local ECM stiffness.

### HT1080 response to type 1 collagen ECM of increasing concentration

In addition to analyzing stiffness around DFs, *κ* was also probed around highly invasive human fibrosarcoma HT1080 cells. Like DFs, HT1080s were cultured in rat tail T1C hydrogels for 48 h prior to AMR measurements. In agreement with past studies^29,36^, isolated HT1080 cells cultured in 3D rat tail T1C hydrogels exhibit rounded morphology (Fig. 1g), preventing identification of a cell front. Thus, *κ* results are represented in a similar way as for DFs, with the exception that stiffness was probed at 0°, 45°, 90°, 135° with respect to the horizontal axis of the image. For purposes of analysis and data presentation, a cell front was arbitrarily assigned as the right side of the cell with respect to the image.

When analyzing all beads in all directions of bead oscillation (Fig. S3d), *κ* around HT1080s was found to be comparable to cell-free stiffness for cells embedded in 1.0T1C (*p* = 0.98), 1.5T1C (*p* > 0.99) and 2.0T1C (*p* = 0.15) hydrogels. Stiffness around HT1080s in 3.0T1C hydrogels was lower than in cell-free 3.0T1C hydrogels (*p* < 0.01), but did not differ from *κ* around cells measured in 2.0T1C hydrogels (*p* = 0.38).

Spatial distribution of *κ* probed at 0° is visualized in Fig. 1h. *κ* probed at other directions is included in Fig. S3a–c. MER of stiffness was conducted as for DF cells and analyzed parameters predicted 42.8% of variance in data (Fig. S3e). T1C concentration was found to be a dominant predictor of stiffness. Distance away from the cell, direction of bead oscillation and angular position $\theta$ were not significant predictors of stiffness. Analysis of the peri-cellular region indicated that stiffness was isotropic in 1.0T1C (*p* = 0.58), 1.5T1C (*p* = 0.94), 2.0T1C (*p* = 0.99) and 3.0T1C (*p* = 0.99) hydrogels.

In order to assess an oscillation-frequency effect on *κ*, AMR was conducted in the peri-cellular region of DFs and HT1080s (< 25 μm, *n*_*beads*_ ≥ 10 per cell) using a wider range of frequencies. AMR data was collected at 20, 50 and 200 Hz at 0° and 90° with respect to long axis of the DFs or with respect to the horizontal axis of the image for HT1080s and cell-free measurements. As observed previously by us^10^ and widely reported by other groups^37–39^, *κ* increased with frequency of bead oscillation, but with the tenfold increase in frequency, *κ* changed on average only by 24% (Fig. S4).

10 beads closest to each cell were also used as probes for passive microrheology (PMR), during which the trapping beam, but not the detection beam, was blocked by a mechanical shutter. Data was recorded for 30 s, with a sampling frequency of 10,000 Hz. Viscoelasticity of hydrogels, reported as the complex valued shear modulus G* = *G′* + *iG″* (Pa), was calculated from the mean-square displacement spectrum of each bead for PMR data^40,41^. For AMR data, G* was calculated from κ* using the generalized Stokes relation^37,42^. *G′* values from PMR were higher than *G″* from PMR (Fig. S5), but significantly lower than *G′* measured by AMR (Fig. S6). In contrast, *G″* values were larger when measured by PMR than by AMR (*p* < 0.01).

### Fibroblast response to stiffness-matched type 1 collagens and fibrin ECMs

**Figure 2:**
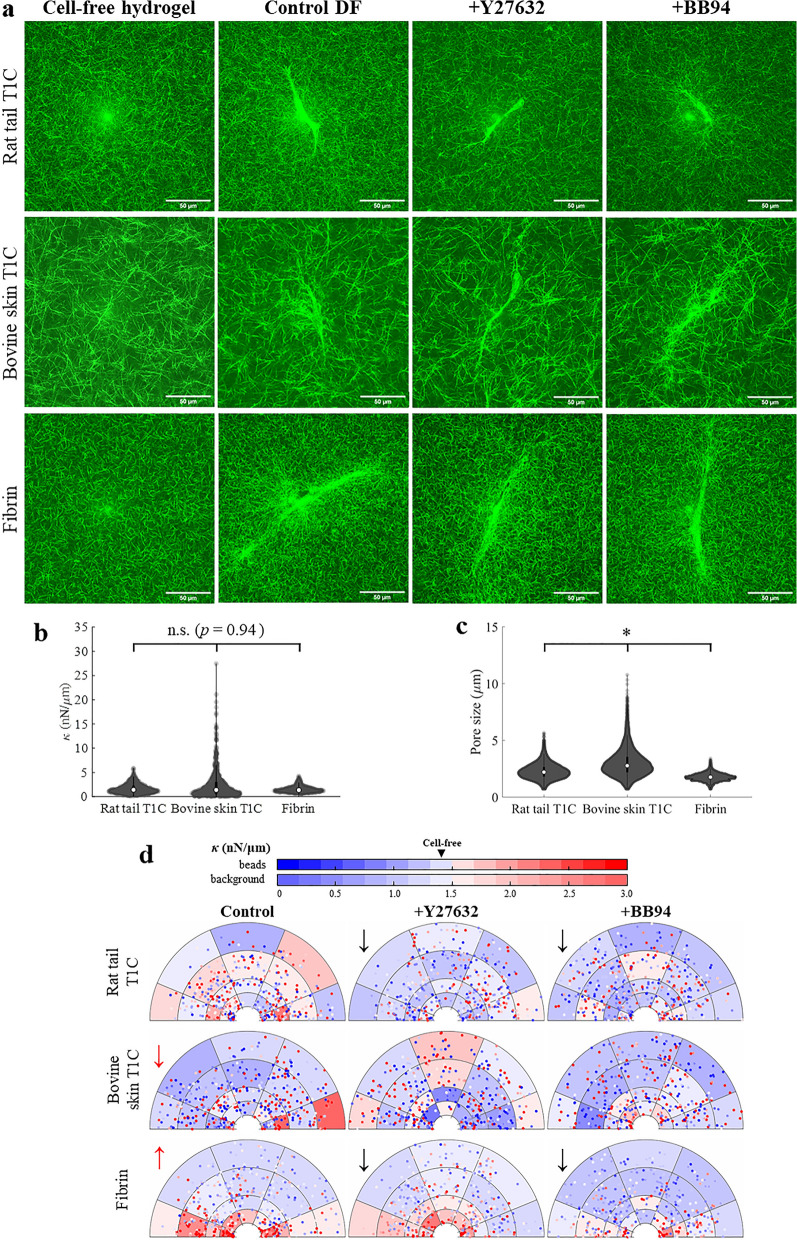
The effect of ECM type and treatment on stiffness distribution around DFs. (**a**) RCM images of cell-free hydrogels (rat tail T1C, bovine skin T1C, fibrin; *n*_*sample*_ = 3 per ECM type, *n*_*beads*_ > 40 per sample) and hydrogels containing DFs either untreated or treated with Y27632 and BB94 (*n*_*sample*_ = 5, *n*_*cells*_ = 10 per condition, *n*_*beads*_ > 40 per cell). (**b**) Stiffness *κ* and (**c**) pore size distribution of cell-free hydrogels. Median values are denoted by white markers. (**d**) *κ* distribution around DFs, probed along long axis of the cell. Background color is shaded according to the median *κ* value in each bin (background color bar). Each data point is a single probed bead, color-coded for *κ* (beads color bar). Arrows indicate significant difference (*p* < 0.05) in *κ*. Control (untreated) conditions are compared to rat tail T1C hydrogels (red arrows), while treatment conditions are compared to the matched control condition (black arrows). Arrow direction indicates increased or decreased stiffness. *p < 0.05.

In the next set of experiments, we assessed the effect of ECM type on peri-cellular stiffness. Instead of matching matrix concentrations, concentrations of fibrin and bovine skin T1C hydrogels were selected to result in median stiffness levels matching the cell-free stiffness of rat tail T1C polymerized at 1.5 mg/ml. Thus, bovine skin T1C hydrogels were prepared at 1.75 mg/ml and fibrin hydrogels were polymerized at 2.7 mg/ml (Fig. 2a). Despite having similar median stiffness values (*p* = 0.94), the dispersion of stiffness values was much greater for bovine skin T1C (*p* < 0.01) and much smaller for fibrin (*p* < 0.01) than for rat tail T1C (Figs. 2b, S1c). Different types of ECM also resulted in distinct fiber architectures. As compared to rat tail T1C hydrogels, median pore size and dispersion of pore size values was greater for bovine skin T1C hydrogels (*p* < 0.01) and lesser for fibrin hydrogels (*p* < 0.01, Fig. 2c). A correlation between *κ* and pore size was not detected.

DFs were shown to regulate their local ECM stiffness differently based on type of hydrogel in which they were cultured in for 48 h. Spatial stiffness distribution probed along the long axis of the cell is shown in Fig. 2d. Results showing *κ* distribution along other directions are included in Fig. S7a–c. MER was used to assess whether type of ECM as well as axis of bead oscillation, the shortest distance between the probed bead and a cell and angular position $\theta$ are significant predictors of *κ* (Fig. S7d). Discrete variables of oscillation axis and ECM type were simply encoded with the reference to cell orientation angle and rat tail T1C, respectively. While analyzed parameters explained only 5% of data variance, *κ* was found to strongly depend on type of ECM. Cells in bovine skin T1C established lower *κ* (*p* < 0.01), and cells in fibrin hydrogels promoted higher *κ* (*p* < 0.01) as compared to *κ* around cells in rat tail T1C or *κ* of cell free hydrogels. In contrast, *κ* around cells cultured in rat tail T1C did not differ from the cell-free stiffness (*p* = 0.96). Distance from the cell profile was also found to be a significant predictor of *κ*. Distance-dependence on *κ* is especially evident when stiffness was probed around DFs cultured in fibrin hydrogels. Peri-cellular stiffening was observed in fibrin and while stiffness decreased with distance away from the cell (*p* < 0.01), stiffening in regions towards cell front and cell back was evident up to 100 μm away from the cell, when beads are oscillated along the long axis of the cell (Fig. 2d, p < 0.01).

Next, the effect of two treatments (Y27632 and BB94) on stiffness landscape around DFs was assessed. Y27632 was previously shown to reduce cell contractility through inhibition of ROCK, while BB94 is a broad spectrum MMP inhibitor^32–34^. Given that both cell contractility and MMP secretion were found to be crucial in cell remodeling of local ECM, addition of either treatment was expected to reduce peri-cellular stiffness, as suggested by our previous studies^9^. However, MER indicated that cell response to treatments varied depending on ECM type (Fig. S7e). As compared to control conditions (no treatment), addition of Y27632 or BB94 resulted in lower *κ* values in rat tail T1C and fibrin hydrogels, but treatment effect was not observed in bovine skin T1C. In rat tail T1C hydrogels, Y27632 or BB94 lowered stiffness to levels below cell-free *κ* (*p* < 0.01), but in fibrin hydrogels BB94 was more effective than Y27632 in reducing peri-cellular stiffening. However, following treatment with either Y27632 or BB94, *κ* in fibrin hydrogels remained higher in the peri-cellular region (< 25 μm, inner annulus) than in the distal region (> 25 μm, *p* < 0.01). Further, only in fibrin hydrogels, *κ* around DFs varied based on axis of bead oscillation (Fig. S7).

**Figure 3:**
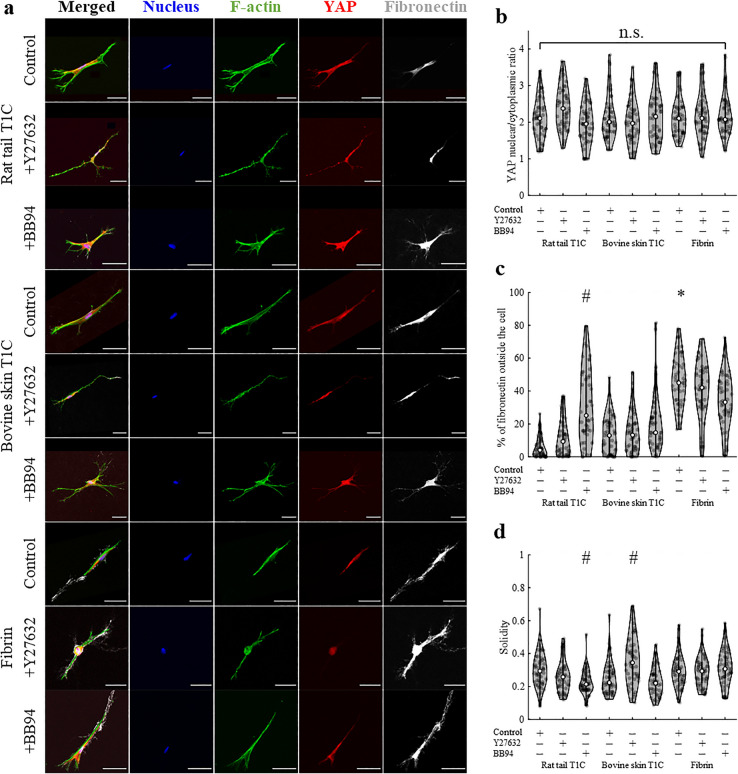
The effect of ECM type and treatment on nuclear/cytoplasmic YAP ratio, fibronectin secretion and solidity of DFs. (**a**) Immunofluorescent confocal images of DFs (*n*_sample_ = 3, *n*_cells_ = 50 per condition). Scale bar = 50 μm. Quantification of (**b**) nuclear/cytoplasmic YAP ratio, (**c**) fibronectin secretion, described as % of fibronectin signal found outside of the cell and (**d**) cell solidity. Statistical difference across tested ECM types (as compared to rat tail T1C) or following Y27632 or BB94 (as compared to control condition) is denoted by * or #, respectively. Detailed statistical comparison between the groups for (**b**–**d**) is shown in Table S1.

In addition to probing local stiffness, confocal microscopy was conducted to image relative expressions and/or locations of F-actin, YAP, fibronectin, and the cell nucleus (Fig. 3a). Despite considerably different *κ* landscapes around DFs cultured in different types of ECM, nuclear/cytoplasmic YAP ratio was found to be comparable for control cells cultured in different types of hydrogels (*p* = 0.77, Fig. 3b, Table S1a). Similarly, addition of either Y27632 or BB94 did not alter nuclear/cytoplasmic YAP ratio as compared to control cells in any type of ECM. Fibronectin secretion, reported as % of total detected fibronectin found outside of the cell, was shown to differ more prominently between the ECM types, but treatment effect was not widely observed (Fig. 3c, Table S1b). In contrast, solidity of DFs, measured as the ratio of cell area to cell convex area, did not differ across the tested types of ECM, but addition of either BB94 or Y27632 altered solidity of cells in collagen hydrogels (Fig. 3d, Table S1c). Nonetheless, fibroblasts exhibited mostly elongated cell morphology, which did not differ greatly between test conditions.

### HT1080 response to stiffness-matched type 1 collagens and fibrin ECMs

**Figure 4:**
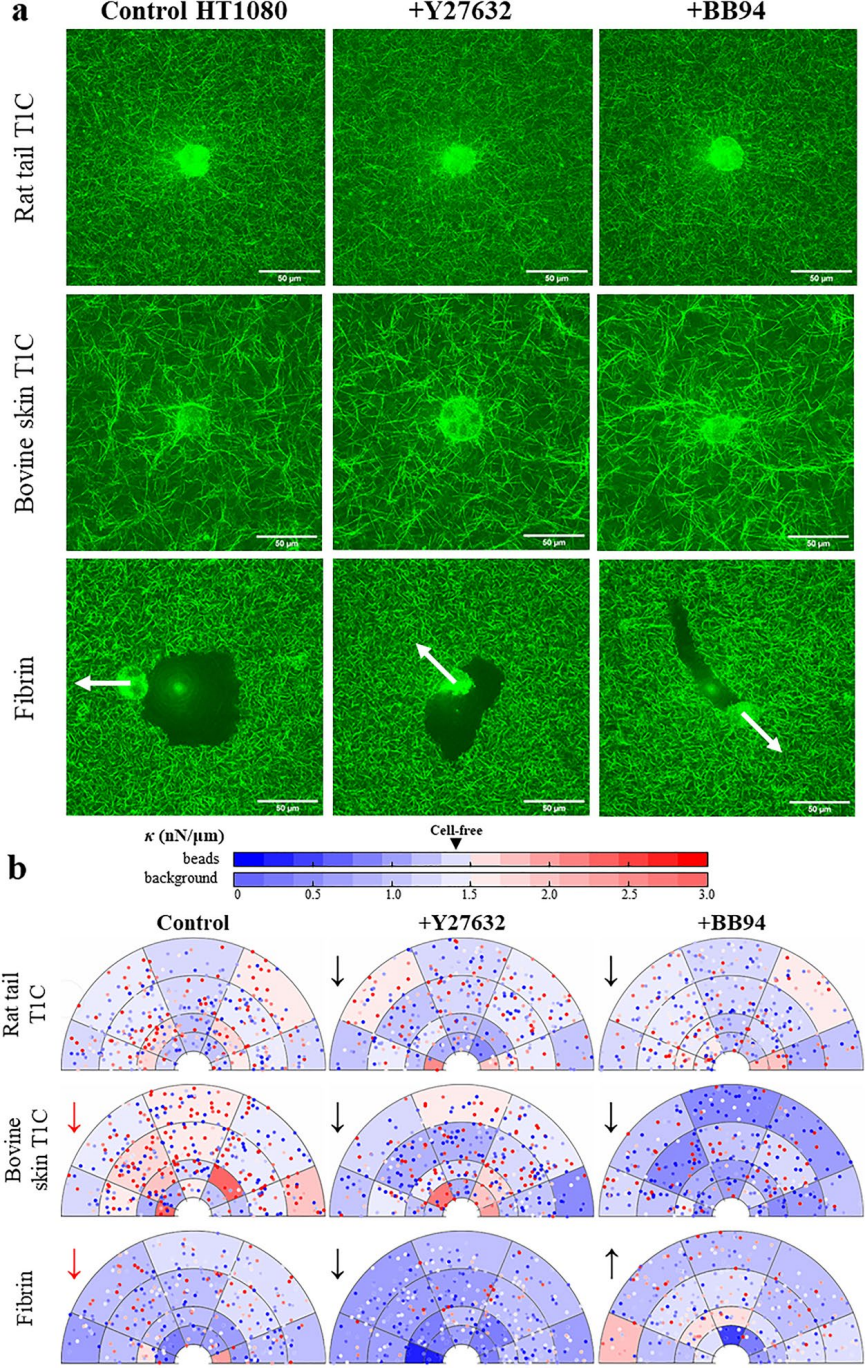
The effect of ECM type and treatment on stiffness distribution around HT1080s. (**a**) RCM images of HT1080 cells cultured in 3 different types of hydrogels (rat tail T1C, bovine skin T1C, fibrin), untreated or treated with Y27632 and BB94 (*n*_sample_ = 5, *n*_cells_ = 10 per condition, *n*_beads_ > 40 per cell). (**b**) *κ* distribution around HT1080s, probed along horizontal axis of the image for cells cultured in rat tail or bovine skin T1C. For cells cultured in fibrin hydrogels, *κ* was probed along the direction closest to the orientation of the enzymatic breakdown, as indicated by the white arrows seen in (**a**). Background color is shaded according to the median *κ* value in each bin (background color bar). Each data point is a single probed bead, color-coded for *κ* (beads color bar). Significant difference in *κ* across tested ECMs (as compared to rat tail T1C, red arrows) or following Y27632 or BB94 treatment (as compared to control condition, black arrows) is marked by arrows indicating increase or decrease in *κ.* *p < 0.05.

Next, fibrosarcoma HT1080 cells were subjected to different types of ECM (Fig. 4a). Of note, significant fibrinolysis was observed when cells were cultured in fibrin hydrogels. Consequently, while stiffness *κ* was still probed at 0°, 45°, 90°, 135° with respect to the image, for cells embedded in fibrin, cell front was assigned away from the region of enzymatic breakdown of fibrin (indicated by arrows in Fig. 4a) instead of arbitrarily assigning cell front to the right side of the cell. Spatial distribution of *κ* along the 0° direction (for collagen hydrogels) or along the direction closest to the orientation of the enzymatic breakdown (for fibrin hydrogels) is shown in Fig. 4b. *κ* measurements along other axes are illustrated in Fig. S8a–c.

MER of local ECM stiffness indicated that culturing HT1080s in bovine skin T1C or in fibrin hydrogels resulted in lower *κ* values as compared to stiffness measured in rat tail T1C hydrogels (Fig. S8d). However, *κ* measured in bovine skin T1C did not differ significantly from *κ* of cell-free hydrogels (*p* = 0.71). Decrease in stiffness was more pronounced in fibrin hydrogels and local *κ* values were shown to be lower towards ECM regions broken down by the cells (towards cell rear).

Treatment effect was observed in all tested types of ECM. Addition of Y27632 or BB94 led to reduction in local *κ* around HT1080s cultured in rat tail T1C and bovine T1C, yet this effect was more evident in bovine T1C hydrogels (Fig. S8e). While Y27632 and BB94 promoted similar stiffness values in rat tail T1C (*p* = 0.17), BB94 reduced *κ* more efficiently than Y27632 in bovine skin T1C hydrogels (*p* < 0.01). In fibrin hydrogels, addition of Y27632 led to significant decrease in *κ* around HT1080s and did not visibly affect fibrinolysis. However, unlike BB94 which was added 48 h before AMR measurements, Y27632 was added only 1 h before AMR measurements. Thus, any effect of Y27632 on fibrinolysis would not be easily discernible. In contrast, addition of BB94 resulted in increase in local *κ*, yet local stiffness was still below the *κ* of cell-free hydrogels (*p* < 0.01) and fibrinolysis was still exhibited by the cells. Only in fibrin hydrogels, distance between the probed bead and cell and angular position $\theta$ (arbitrary for HT1080s in collagen hydrogels) were found to be significant predictors of *κ*. *κ* was found to increase further away from the cell and away from the region broken down by fibrinolysis.

**Figure 5:**
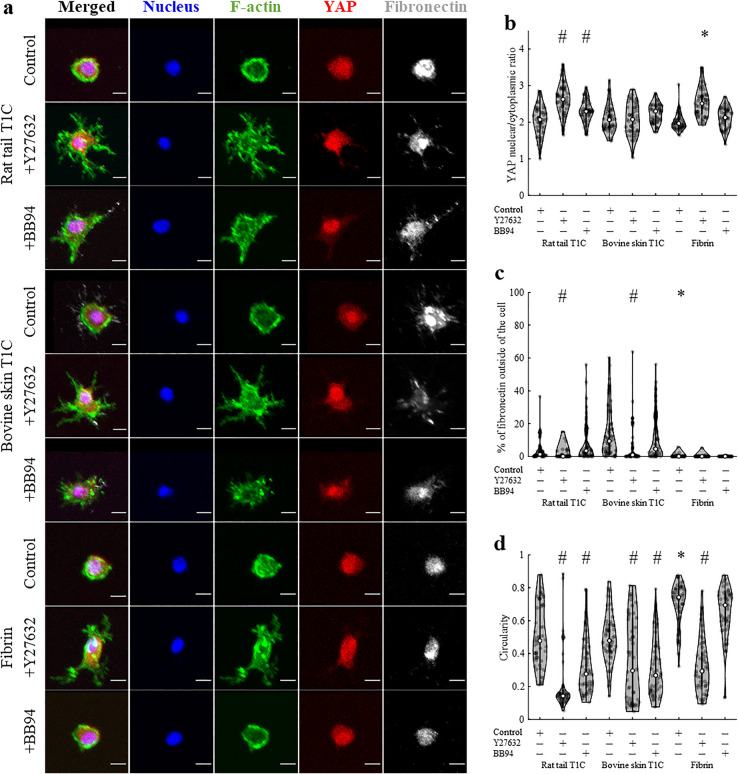
The effect of ECM type and treatment on nuclear/cytoplasmic YAP ratio, fibronectin secretion and circularity of HT1080s. (**a**) Immunofluorescent confocal images of HT1080s (*n*_sample_ = 3, *n*_cells_ = 50 per condition). Scale bar = 10 μm. Quantification of (**b**) nuclear/cytoplasmic YAP ratio, (**c**) fibronectin secretion, described as % of fibronectin signal found outside of the cell and (**d**) cell circularity. Statistical difference across tested ECM types (as compared to rat tail T1C) or following Y27632 or BB94 (as compared to control condition) is denoted by * or #, respectively. Detailed statistical comparison between the groups for (**b**–**d**) is shown in Table S2.

ECM type and tested treatments altered not only local *κ* landscapes, but also affected various cell properties (Fig. 5a, Table S2). Nuclear/cytoplasmic YAP ratio did not differ for control cells cultured in different types of hydrogels, but differed following treatments for cells in rat tail T1C or in fibrin (Fig. 5b). While the extent of fibronectin secretion (Fig. 5c) and cell circularity (Fig. 5d) was comparable for cells cultured in different types of collagen, fibronectin secretion decreased and circularity increased when cells were cultured inside fibrin hydrogels. Addition of either Y27632 or BB94 promoted more dendritic morphology of HT1080s, but treatment effect on other nuclear/cytoplasmic YAP ratio and fibronectin secretion varied with type of ECM (Table S2).

## Discussion

Stiffness around cells was previously shown by our group to depend on T1C concentration^11^ and vary with different treatments and between tested cell lines^10,43^. Here, we add new information about stiffness and its anisotropy around two cell lines cultured in distinct ECMs, varying in source, porosity and concentration. For all conditions, *G′* values were significantly lower when calculated from PMR data than when estimated from AMR data (Fig. S6). These results are in agreement with previously reported data that found passive microrheology to underestimate *G′* due to lower signal-to-noise ratio and the assumption of thermal equilibrium that does not account for the influence of the optical trap and external forces from the cells in calculations of the G* modulus^37,42^. Further, the calculation of G* assumes the material is a local continuum, and that pore size is considerably smaller than the probe bead^37,42^, which is not the case for rat tail 1.0T1C, 1.5T1C and 2.0T1C hydrogels. However, in cell-free 3.0T1C hydrogels (Fig. 1b), the pore size distribution shows that most pores are smaller than the bead diameter (2 μm). This crossing of spatial scale may influence the interpretation of AMR data, and may explain in part the similarity in stiffness distribution between 2.0T1C and 3.0T1C hydrogels.

Stiffness around DFs and HT1080s was probed in 4 distinct directions elucidating local anisotropies. Stiffness and cell properties, including expressed nuclear/cytoplasmic YAP ratio, cell solidity or circularity and percentage of secreted fibronectin were shown to vary across the tested ECM types and treatments, indicating a complex cell-ECM relationship based on a variety of factors and characteristics of both cells and the ECM. ECM concentration was found to be a dominant predictor of local stiffness for DFs and HT1080s cultured at different concentrations of rat tail T1C (Figs. 1, S2, S3). These findings are seemingly in opposition to our past studies that found peri-cellular stiffness to be comparable around DFs cultured for 24 h in rat tail 1.0T1C or 1.5T1C hydrogels^10^. Peri-cellular stiffening observed 24 h after hydrogel preparation was not observed in the current study at the 48 h time point. Further, in contrast to our previous studies^10^ and findings by Loeber et al. for chondrocytes cultured in a hyaluronic acid gel^44^, stiffness was largely unaffected by distance from the cell, angular position *θ*, and axes of bead oscillation. Discrepancy in results could potentially stem from the difference in duration of cell culture, as supported by past research that found hydrogel stiffness to vary with cell incubation time^45,46^. Reported results are most likely by alternative factors not explored in this project,, including cell seeding density^46^, difference in collagen composition^47^, cell area^48–50^ and expression levels of mesenchymal proteins including those for actin, actin polymerization, myosin motors as well as the state of regulatory proteins including ROCK.

Both DFs and HT1080s were also shown to respond differently when cultured in three distinct types of hydrogels, formulated to have comparable cell-free median stiffness (Fig. 2b). Our results indicate that cell response to an ECM might not be governed by median stiffness levels alone. Past studies found that cells sense local stiffness anisotropies in 3D hydrogels^51^ and thus, cells might also be sensitive to the magnitude of local variances in stiffness within the hydrogels (Fig. 2b). Further, all three types of hydrogels exhibited distinct porosities and microarchitectures—factors, which are known to significantly affect cell survival, proliferation, and migration^52–54^. In addition to detecting differences in mechanical properties of hydrogels, cell behavior is known to vary with biochemical properties of the ECM^1,55–57^, which is also corroborated by our study. The impact of ECM type on cell properties and local stiffness levels was most pronounced when comparing data collected in collagen hydrogels and fibrin hydrogels. For example, while DFs promoted peri-cellular stiffening in fibrin as compared to either rat tail or bovine skin T1C (Fig. 2d), HT1080s prominently degraded local fibrin, but not collagen matrix (Fig. 4a)^58–60^. Even though stiffness levels increased following BB94 treatment of HT1080s in fibrin, fibrinolysis was still observed (Fig. 4). This observation could be consistent with the molecular action of BB94, which is a broad spectrum inhibitor of zinc MMPs, whereas the enzymatic breakdown of fibrin by HT1080s is associated with expression of serine proteases and not directly with MMP activity^61,62^.

Rat tail T1C and bovine skin T1C were prepared using the same protocol and differed only with the tissue source of telocollagen. Based on results of SPS-Page tests available from the manufacturer (see reports in Supplementary Information), both types of collagen exhibited similar purity with over 85% of T1C contained within contained within α, β and γ bands. Nonetheless, discrepancy in fiber architectures between the two sources of collagen (Fig. 2a,b) could potentially stem from small differences in amino acid compositions, presence of distinct collagen subtypes other than type 1 or different fibrillogenesis dynamics, which were all previously shown to differ with collagen source, including tissue type and species^63–65^. In our study, cells embedded in bovine skin T1C hydrogels with larger pore sizes established lower stiffness values than cells cultured inside rat tail T1C hydrogels with smaller pores (Fig. 2b). These findings indicate that local ECM stiffness established by the cells decreases with increasing pore size. However, the relationship may not be causal because the cells can also respond to biochemical differences between the collagen types^36^. While cells were shown to differentially respond to different types of ECM, the small predictive power of MER suggests that a more comprehensive analysis of factors governing peri-cellular stiffness is still required.

Despite observed effect of ECM type and treatment on stiffness around cells, change in nuclear/cytoplasmic YAP was only detected for Y27632 treatment of HT1080 cells (Fig. 4b, Table S2). While YAP expression was shown to be more prominent inside the nuclei than inside cell cytoplasm for all tested conditions, YAP signal was still widely distributed throughout each cell. Translocation of YAP to the nucleus has been widely reported for cells cultured on 2D substrates. While the exact substrate stiffness at which translocation occurs is difficult to determine and varies with tested cell line, substrate type or added treatment^66–69^, translocation is commonly reported when substrate stiffness exceeds 1 kPa^13,70,71^. For 3D cultures, YAP translocation into the nucleus also varies with cell and ECM type^71–73^. For example, past studies on fibroblasts embedded inside synthetic fibrous hydrogels reported increase in nuclear/cytoplasmic YAP ratio with fiber density^72^, indicating a role in mechanotransduction, but mechanotransduction of human breast cancer cells in 3D cultures was found to be independent of YAP^73^. A lack of Y27632 treatment effect on YAP ratio in this manuscript is also in agreement with studies on macrophages cultured on glass and fibrin hydrogel substrates^74^ or fibroblasts cultured in 3D collagen hydrogels^75^, which similarly reported no change in YAP ratio with Y27632 treatment. We assert that a further understanding of the role of YAP in mechanosensing requires measurements of local peri-cellular and not bulk stiffness of the ECM. Such studies may clarify the signal-to-stiffness relationship. Our findings presented here do measure the stiffness sensed by the cells and provide new, but far from comprehensive understanding regarding roles of ECM types and tested treatments on YAP ratio. Despite no prominent difference in nuclear/cytoplasmic YAP between analyzed conditions, lack of change in YAP ratio could also be attributed to a narrow range of tested stiffnesses in our study (*G′* = 0.1–1000 Pa), perhaps preventing more prominent YAP translocation to nuclei in stiffer hydrogels or to cytoplasm in softer hydrogels.

Comparatively, fibronectin secretion was shown to be more correlated with local stiffness (Figs. 3c, 5c). For instance, DFs exhibited highest peri-cellular stiffness and fibronectin secretion inside fibrin hydrogels as compared to collagen hydrogels. In contrast, HT1080s promoted much lower stiffness levels and fibronectin secretion inside fibrin hydrogels than inside collagen hydrogels. Nonetheless, after 48 h of cell culture, fibronectin expression, considered to be colocalized with newly secreted collagen^76–78^, was not prominent in the extracellular space. Most of the fibronectin signal was detected inside the cells (Fig. 3a), which is in agreement with past studies on fibroblasts in fibrin hydrogels after 48 h of culture^79,80^. Similarly, collagen secreted by fibroblasts cultured in collagen hydrogels was previously found to be limited to the cell perimeter after 48 h of culture and was present throughout the whole hydrogel only after 12 days of culture^81^. While DFs promote formation of fibronectin fibrils^79,80^, HT1080s possess limited ability to assemble fibronectin fibrils without dexamethasone stimulation^82,83^. In agreement with past research, our study shows that fibronectin secretion by HT1080s was largely non-fibrillar and lesser in extent than observed for DFs (Fig. 5a). Extent of fibronectin expression indicates that ECM probed by AMR was composed of mostly original, not cell-secreted, ECM. Nonetheless, trends in stiffness across tested ECMs were shown to follow trends in fibronectin expression, and further studies are required to explicate the relationship between peri-cellular stiffness and ECM secretion by cells.

Differential effect of ECM type on how cells remodel the matrix is further evidenced by the addition of Y27632 or BB94 treatments. Stiffening of local matrix by the tested cell types is known to be mediated by contractile forces, which can be inhibited by Y27632^9,10,84^ and local matrix degradation by MMP secretion, which is inhibited by BB94^32–34^. Interestingly, past studies by our group have showed that MMP secretion can also contribute to stiffening of the ECM, most likely by allowing cell elongation within a dense ECM^9^. In fact, MMP activity and cell contractility were essential to ECM stiffening for the case of dermal fibroblasts and aortic smooth muscle cells in type 1 collagen^9^. Results from our current study support this finding across multiple ECM types (Figs. 2d, 4b, S7, S8), and further show that Y27632 and BB94 treatments also alter morphology of both cell types, yet the effect of Y27632 and BB94 on YAP and fibronectin expression varied with the type of ECM (Figs. 3, 5).

In conclusion, this work provides further evidence of the importance of measuring peri-cellular and not only bulk properties of the ECM when exploring biophysics-based hypotheses in cell biology. Our group has repeatedly observed that bulk stiffness might not reflect stiffness sensed by the cells and assert peri-cellular measurements should be included in comprehensive studies on cellular mechanotransduction^9,10,85^. In this work we observed a complex relationship between stiffness established by dermal fibroblasts or HT1080 fibrosarcoma cells and ECM properties, such as hydrogel concentration, type, fiber architecture and pore size. While tested cell lines created highly heterogeneous stiffness landscapes, the cell metrics under investigation, namely ECM remodeling (assessed by stiffness), YAP localization, fibronectin secretion and shape did not vary with the initial concentration of rat tail T1C hydrogel. In contrast, cells responded differentially when embedded inside type 1 collagen ECMs of different origin, but with matched initial median stiffness. These types of results indicate that we, and others, are excluding important biophysical dimensions in our studies of cellular mechanotransduction, and that the ECM must be characterized in multiple ways (e.g. stiffness distribution, architecture, fiber size, porosity, persistence length) before we can better understand its influences on cells. Importantly, we acknowledge we have just scratched the surface of measuring cell responses and future studies should include measures of gene expression down mechanosensing and extracellular protein synthesis pathways, and should likely explore the adhesome, which may explain difference in cell behavior in stiffness-matched matrices. The future addition of real-time imaging of adhesion and membrane dynamics, calcium signaling, and matrix deformation will complement the observed complex mechanical landscapes in helping us to understand precisely how local stiffness values, and their changes on the micron scale, can instruct cells.

## Materials and methods

### Cell culture

Normal human dermal fibroblasts (DFs, Lonza) were cultured in Dulbecco's Modified Eagle's Medium (DMEM) with low glucose, l-glutamate, and sodium pyruvate (ThermoFisher) and supplemented with 10% Fetal Bovine Serum (FBS, Gibco) and 1% penicillin streptomycin (Gibco). Human fibrosarcoma HT1080 cells (ATCC) were cultured in Eagle's Minimum Essential Medium (EMEM, ATCC) supplemented with 10% FBS and 1% penicillin streptomycin.

### Collagen hydrogel preparation

Rat tail collagen hydrogels were prepared at 1.0, 1.5, 2.0 and 3.0 mg/ml concentrations using type I rat tail telocollagen (Advanced Biomatrix, #7858). Bovine skin collagen hydrogels were prepared at 1.75 mg/ml concentration using type I bovine skin telocollagen (Advanced Biomatrix, #7811). Following protocol by Doyle^86^, collagen was supplemented with 10X Phosphate-Buffered Saline (PBS, ThermoFisher), 10X DMEM (Sigma), 10X reconstitution buffer^86^, 1 N NaOH (ThermoFisher), 2 μm carboxylated silica microbeads (0.8 mg/ml, Bangs Laboratories), and cells (50 k/ml). Hydrogels were polymerized inside 35 mm glass bottom dishes (MatTek) in a standard tissue culture incubator at 37 °C and 5% CO_2_. After 30 min, hydrogels were hydrated with 2 ml of culture media supplemented with 25 mM HEPES (Gibco). Treatment of 20 μM Y27632 (Sigma, #0000097404) or 10 μM BB94 (Sigma, #3560212) was added to media 1 h or 48 h before AMR measurements, respectively.

### Fibrin hydrogel preparation

Fibrin hydrogels were prepared at 2.7 mg/ml concentration as previously described^43,87^. Bovine stock fibrinogen (Sigma, SLCG6303) was dissolved in PBS, filtered and supplemented with 2 μm carboxylated silica microbeads and 50 k/ml of cells. 1 ml hydrogel was polymerized inside of a 35 mm glass bottom dish (MatTek), following the addition of bovine thrombin (4 U/ml, Sigma, SLBW2056). Hydrogels were incubated in a standard tissue culture incubator at 37 °C and 5% CO_2_ for 30 min and then hydrated with 2 ml of culture media supplemented with 25 mM HEPES (Gibco). Treatment of 20 μM Y27632 or 10 μM BB94 was added to media 1 h or 48 h before AMR measurements, respectively.

### Microstructural assessment of hydrogels

Cell-free hydrogels were prepared 48 h before imaging and stained with Atto 488 NHS ester dye (Sigma). Fluorescent images were obtained with the 488 nm laser line using Fluoview3000 laser scanning microscope equipped with a UPlanSApo 40x/1.25 NA silicone immersion objective lens (Olympus). Z-stacks of images were collected between 30 and 50 µm from the glass height using a step size of 0.25 µm, a scanning speed of 2 µs/pixel and a total scan resolution of 1024 pixels × 1024 pixels across a 96.72 µm × 96.72 µm field-of-view (FOV).

Porosity of cell-free hydrogels was assessed using deconvoluted and binarized images from 6 FOVs per hydrogel obtained from 3 distinct hydrogels per condition. Pore size in cell-free hydrogels was calculated as the maximum diameter of a sphere inscribed inside each pore. Individual pores were identified using Distance Transport Watershed 3D algorithm and quantified using Analyze Regions 3D algorithm, both of which are incorporated into MorphoLibJ plug-in^88^ for Fiji software^89^.

### Active and passive microrheology

Stiffness was measured using multi-axes optical tweezers active microrheology system previously described in^10^ and^87^. Briefly, each hydrogel sample was mounted in a dish holder placed inside the stage-top nanopositioning piezoelectric stage (P-545.xR8S PInano XYPiezoSystem,PI). Individual microbeads were oscillated by applying optical forces applied with a continuous-wave fiber laser with an emission at 1064 nm (YLR-5–1064-LP, IPG Photonics). Trapping beam oscillations with an amplitude of 60 nm and frequency of 50 Hz, unless specified otherwise, were produced by the movement of a pair of galvanometer mirrors (GVS012, ThorLabs), located conjugate to the back focal plane of the microscope objective lens (60x-oil PlanApo TIRFM 1.45NA, Olympus). A detection laser beam of wavelength 785 nm generated by a single mode fiber-pigtailed laser diode (LP785-SF100, ThorLabs) was co-aligned with the trapping beam at the center of the bead. Change in bead position and trapping beam position were recorded by two quadrant photodiodes (detQPD and trapQPD, 2901 and 2903, respectively, Newport) and used to calculate a complex material response (${\alpha }^{*}(\omega)$) by the relationship X= ${\alpha }^{*}(\omega)$ *F,* where *X* and *F* are the Fourier components of bead displacement and optical force, respectively. ${\alpha }^{*}\left(\omega\right)$ is computed once for each oscillation direction under the assumption that α*$(\omega)$ oscillates purely along that axis. Reported stiffness *κ′(ω)* represents the real component of inverse α*. Imaginary component of κ*(ꞷ) was found to be much smaller than the real component, indicating a weak effect of viscous dissipation and it is thus not reported in this study. Using generalized Stokes relation, κ*(ꞷ) can be used to calculate complex shear response ${G}^{*}(\omega)$, which is represented as ${G}^{*}(\omega)=G^{\prime}(\omega)+G^{\prime \prime} (\omega)= (\omega)/6\pi r$, where *r* is the radius of the bead (1 µm)^37,42^. The proportionality parameter relating detQPD signals to bead displacements was measured in situ per bead and per angle of oscillation. After centering the stage on each bead, the stage was moved 200 nm across the bead with a constant velocity of 100 nm/s. Recorded detQPD voltages were used to quantify the voltage-to-μm conversion factor^90–92^, which was later used to calculate bead displacement during AMR measurements. Prior to AMR measurements in hydrogels, the AMR system was calibrated in water, as previously described^9,10,37^. 10 cells across 5 samples were analyzed per condition. At least 40 beads were probed around each cell and each probed bead was located approximately 35 µm from the cover glass and oscillated along 4 different directions. In cell-free hydrogels and around HT1080s, beads were oscillated at 0°, 45°, 90° and 135° with respect to the horizontal axis of the camera image FOV. Around DFs, beads were oscillated at 0°, 45°, 90° and 135° with respect to the long axis of the cell. All reported AMR measurements were found to be above our limit of detection^51^. In addition to AMR measurements, passive microrheology (PMR) data was collected by recording detQPD signals for 30 s with only detection laser beam positioned at the center of the bead. *G′* and *G″* values were calculated from PMR data using the code developed by the Kilfoil lab at University of Massachusetts at Amherst^40^.

### Immunostaining

HT1080 and DF cells were cultured for 48 h inside collagen hydrogels prepared in 12 well glass bottom plates (Cellvis) or inside fibrin hydrogels prepared in 35 mm glass bottom dishes (MatTek). Cells were then fixed in 4% paraformaldehyde (PFA, VWR) for 10 min at room temperature (RT), washed three times with PBS and permeabilized using 0.3% Triton X-100 (Sigma) diluted in PBS. Afterwards, cells were incubated in anti-YAP1 (G6) antibody (sc-376830, Santa Cruz Biotechnologies) for 1 h at RT, washed with 2% bovine serum albumin (BSA, VWR) diluted in PBS and incubated with secondary Alexa Fluor 594 Goat anti-Mouse IgG (BioLegend) antibody for another hour at RT. Subsequently, cells were washed with BSA, incubated with anti-fibronectin antibody (F3648, Sigma) for 1 h at RT, washed again with 2% BSA and incubated with secondary Cyanine5 Goat anti-Rabbit IgG (Invitrogen) antibody for 1 h at RT. Nuclei and F-actin were stained using NucBlue™ Live ReadyProbes™ Reagent (Hoechst 33342, Invitrogen) and Alexa Fluor 488 Phalloidin (Invitrogen) diluted in 2% BSA in PBS for 30 min at RT, respectively. Lastly, hydrogels were washed with PBS and covered with Fluoromount-G (Invitrogen).

### Confocal microscopy and image analysis

Prior to AMR measurements, brightfield image of each cell was acquired using an EO-4010 Monochrome USB 3.0 Camera (Edmund Optics) incorporated in our AMR system and analyzed as described previously^10^. Briefly, images were processed in MATLAB (The MathWorks Inc.) using the image processing toolbox. The cell orientation was quantified by manually tracing the outline of the cell and using the *regionprops* function in MATLAB. After AMR measurements, the shortest distance between the pixel location of each probed bead and the manually traced cell shape cell was calculated in MATLAB and converted into micrometers. Angular position $\theta$ from 0° (cell front) to 180° (cell rear) relative to the long axis of the cell was found by calculating the angle between the pixel position of the bead and centroid of the cell.

Following AMR measurements, brightfield and reflection confocal microscopy (RCM) images of the probed FOV were collected using the 488 nm laser of the Fluoview 1200 laser scanning confocal microscope (Olympus) with the microscope objective lens used for AMR. For DFs, brightfield images collected after AMR measurements were compared with brightfield images collected after AMR to identify the direction of cell migration and, consequently, cell front and cell rear. Beads distal to the cell did not move and serve as reliable fiducial markers. The direction of cell migration was verified by observing change in cell position with respect to these distal beads. In contrast, HT1080s did not migrate visibly during data acquisition and had more circular morphology, with no clear leading and trailing edge. Thus, for purpose of analysis, cells were assumed to migrate towards the right direction with the exception of HT1080s cultured in fibrin that were assumed to migrate away from the ECM region degraded by fibrinolytic processes.

Immunostained cells were imaged one at a time using Fluoview3000 laser scanning microscope equipped with a UPlanXApo 10X/0.40 NA objective lens (Olympus). Z-stacks of images were collected with a step size of 1–2 µm, a scanning speed of 2 µs/pixel and a total scan resolution of 3.22 pixels per micron across area occupied by the cell. For each condition, 50 cells across 3 samples were imaged. Z-stacks of images were first analyzed in Fiji software^89^ to find maximum intensity projection (MIP) across all image planes. MIP images were then processed using custom code written in MATLAB that masked the nuclei, F-actin and fibronectin regions. Nuclear/cytoplasmic ratio of YAP was calculated by dividing mean intensity of YAP inside the nuclei by mean intensity of YAP inside the cytoplasm colocalized with F-actin mask. Proportion of fibronectin found inside the cell was found by calculating the percentage of colocalization of fibronectin mask within F-actin mask. Cell morphological features including cell circularity calculated as *(4·Area·π)/(Perimeter)*^*2*^ and solidity computed as *Area/Convex Area*^93^ were analyzed using F-actin mask and *regionprops* function in MATLAB.

### Statistical analysis

The effect and predictive power of various parameters on stiffness distribution around the cells was assessed using multivariate exponential regression (MER) conducted in R software^35,94^. All other statistical tests were performed in MATLAB. Comparison of non-normally distributed (*p* < 0.01, Kolmogorov–Smirnov test) stiffness values and parameters obtained from image analysis was conducted using non-parametric statistical analyses at a significance level of 0.05. The Friedman test was used to compare stiffness measurements between each oscillation axes. The Kruskal–Wallis test was performed for comparison of multiple groups. The post-hoc Tukey–Kramer test was used to compare specific groups. The Pearson correlation coefficient (ρ) was used to quantify the correlation between stiffness and frequency of bead oscillation. ρ was also used to quantify correlations between pore size and stiffness. The Ansari-Bradley test was used to compare differences in spread of data across tested types of ECM.

## Supplementary Information

Supplementary Information.

## Data Availability

The data that support the findings of this study are available from the corresponding author upon reasonable request.
