# Supplementary material for: Cell mediated remodeling of stiffness matched collagen and fibrin scaffolds
Source: Sci Rep. 2022 Jul 11;12:11736. doi: 10.1038/s41598-022-14953-w (PMC9273755; doi:10.1038/s41598-022-14953-w)
Supplement: Supplementary file 1 — Supplementary Information. [file 41598_2022_14953_MOESM1_ESM.pdf]

a

	Angle of oscillation	1.0T1C	1.5T1C	2.0T1C	3.0T1C
Median ± IQR κ (nN/μm)	All angles	0.61 ± 0.63	1.41 ± 1.29	2.51 ± 1.64	2.72 ± 0.86
	0°	0.61 ± 0.61	1.47 ± 1.43	2.43 ± 1.39	2.82 ± 0.97
	45°	0.62 ± 0.59	1.47 ± 1.21	2.60 ± 1.58	2.67 ± 0.87
	90°	0.58 ± 0.68	1.33 ± 1.29	2.59 ± 1.75	2.66 ± 0.85
	135°	0.60 ± 0.63	1.44 ± 1.29	2.60 ± 1.71	2.72 ± 0.74
n_{beads}		128	141	153	149
$n_{samples}$		3	3	3	3
Kruskal-Wallis test		$p = 0.84$	$p = 0.96$	$p = 0.44$	$p = 0.57$

b

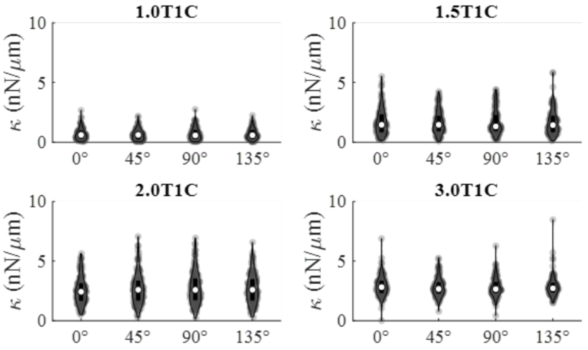

c

	Angle of oscillation	Rat tail T1C	Bovine skin T1C	Fibrin
Median ± IQR κ (nN/μm)	All angles	1.41 ± 1.29	1.39 ± 2.39	1.36 ± 0.69
	0°	1.47 ± 1.43	1.52 ± 1.93	1.41 ± 0.75
	45°	1.47 ± 1.21	1.42 ± 2.69	1.36 ± 0.70
	90°	1.33 ± 1.29	1.46 ± 2.50	1.36 ± 0.74
	135°	1.44 ± 1.29	1.31 ± 2.40	1.31 ± 0.69
n_{beads}		141	186	138
$n_{samples}$		3	3	3
Kruskal-Wallis test		$p = 0.96$	$p = 0.99$	$p = 0.82$

Figure S1. Stiffness κ probed inside cell-free T1C hydrogels. Median ± IQR κ values (a) and κ distributions (b) for cell-free hydrogels polymerized at 4 different concentrations and probed along 4 distinct axes. (c) Median κ values for 3 different types of hydrogels (rat tail T1C, bovine skin T1C, fibrin) polymerized at concentrations resulting in similar stiffness values. Isotropy of cell-free hydrogels is assessed using the Kruskal-Wallis test (a,c).

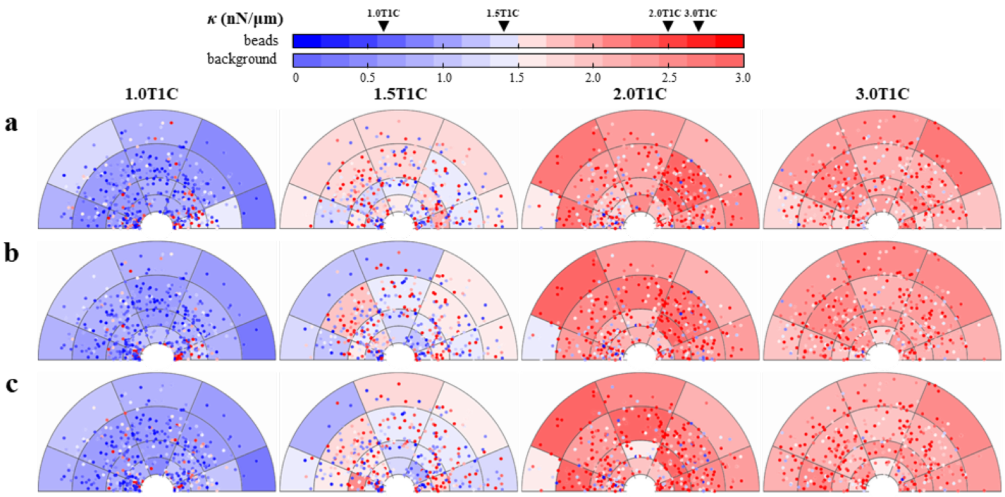

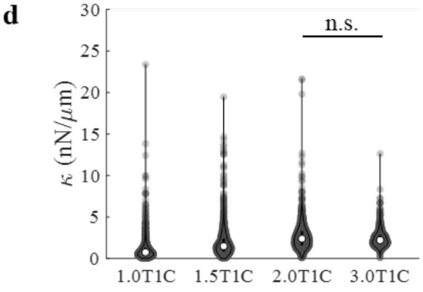

Median ± IQR κ (nN/μm)	0.75 ± 0.79	1.49 ± 1.44	2.38 ± 1.63	2.21 ± 1.01
n_{beads}	451	403	419	415
n_{cells}	10	10	10	10

e **MER summary table**

$$\kappa = (e^{\beta_0})(e^{\beta_1 \text{distance}})(e^{\beta_2 \theta})(e^{\beta_3 \text{axis}_{+45} + \beta_4 \text{axis}_{+90} + \beta_5 \text{axis}_{+135}})(e^{\beta_6 1.5\text{T1C} + \beta_7 2.0\text{T1C} + \beta_8 3.0\text{T1C}})$$

Adjusted R^2: 0.3148

Coefficients:	Estimate	Std. Error	p
(intercept)	-0.30 (β_0)	2.86e-2	< 0.01
distance	-2.15e-6 (β_1)	2.42e-4	0.99
θ	-4.52e-4 (β_2)	1.49e-4	< 0.01
oscillation axis (+45°)	1.49e-2 (β_3)	2.39e-2	0.53
oscillation axis (+90°)	2.55e-2 (β_4)	2.39e-2	0.29
oscillation axis (+135°)	2.46e-2 (β_5)	2.39e-2	0.30
concentration (1.5T1C)	0.66 (β_6)	2.38e-2	< 0.01
concentration (2.0T1C)	1.13 (β_7)	2.37e-2	< 0.01
concentration (3.0T1C)	1.12 (β_8)	2.40e-2	< 0.01

Figure S2. Supplementary material to Figure 1f. Stiffness κ distribution around DFs probed at (a) +45°, (b) +90° and (c) +135° with respect to the long axis of the cell. (d) κ distributions around DFs cultured in rat tail T1C polymerized at 4 different concentrations. Plots aggregate κ probed in all 4 directions. (e) MER summary table. Discrete variables of oscillation axis and concentration were simply encoded with the reference to cell orientation angle and 1.0T1C concentration.

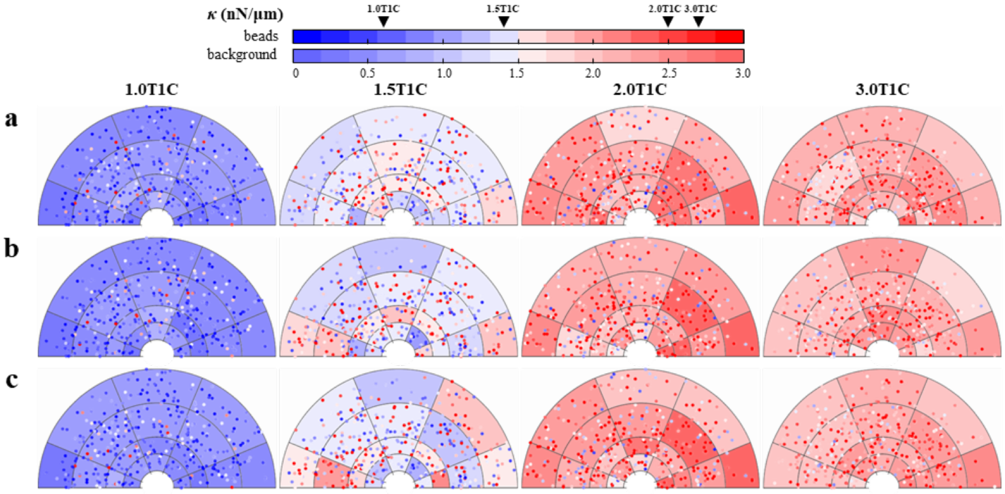

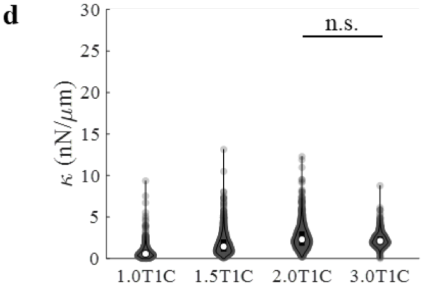

Median ± IQR κ (nN/μm)	0.57 ± 0.63	1.43 ± 1.36	2.29 ± 1.61	2.11 ± 0.86
n_{beads}	404	421	404	417
n_{cells}	10	10	10	10

e **MER summary table**

$$\kappa = (e^{\beta_0})(e^{\beta_1 \text{distance}})(e^{\beta_2 \theta})(e^{\beta_3 \text{axis}_{45} + \beta_4 \text{axis}_{90} + \beta_5 \text{axis}_{135}})(e^{\beta_6 1.5\text{T1C} + \beta_7 2.0\text{T1C} + \beta_8 3.0\text{T1C}})$$

Adjusted R^2: 0.4284

Coefficients:	Estimate	Std. Error	p
(intercept)	-0.60 (β_0)	2.97e-2	< 0.01
distance	-1.98e-4 (β_1)	1.97e-4	0.31
θ	-7.03e-5 (β_2)	1.53e-4	0.65
oscillation axis (45°)	8.54e-3 (β_3)	2.24e-2	0.70
oscillation axis (90°)	2.74e-3 (β_4)	2.24e-2	0.90
oscillation axis (135°)	2.64e-2 (β_5)	2.24e-2	0.24
concentration (1.5T1C)	0.92 (β_6)	2.24e-2	< 0.01
concentration (2.0T1C)	1.40 (β_7)	2.27e-2	< 0.01
concentration (3.0T1C)	1.35 (β_8)	2.25e-2	< 0.01

Figure S3. Supplementary material to Figure 1h. Stiffness κ distribution around HT1080s probed at (a) 45°, (b) 90° and (c) 135° with respect to the horizontal axis of the image. (d) κ distributions around HT1080s cultured in rat tail T1C polymerized at 4 different concentrations. Plots aggregate κ probed in all 4 directions. (e) MER summary table. Discrete variables of oscillation axis and concentration were simply encoded with the reference to horizontal axis of the image and 1.0T1C concentration.

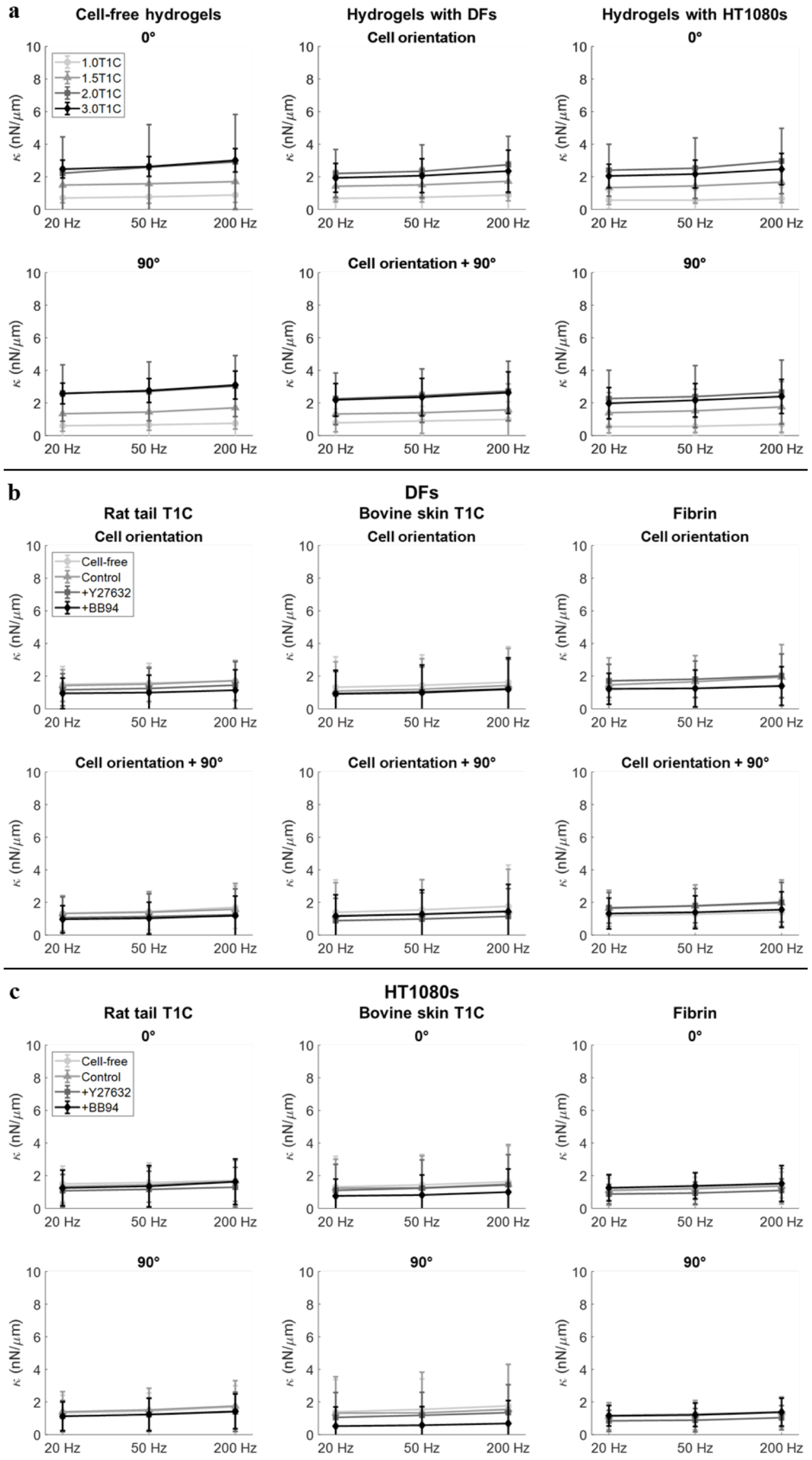

Figure S4. Stiffness κ measured at different frequencies of bead oscillation (median ± IQR). κ was probed along and across the long axis of the cell (for DF cells) or along horizontal and vertical axis of the image (for HT1080s and cell-free hydrogels). Median κ values for (a) rat tail T1C hydrogels polymerized at different concentrations and probed around (b) DFs and (c) HT1080s cultured in 3 different types of hydrogels (rat tail T1C, bovine skin T1C, fibrin), untreated or treated with Y27632 or BB94.

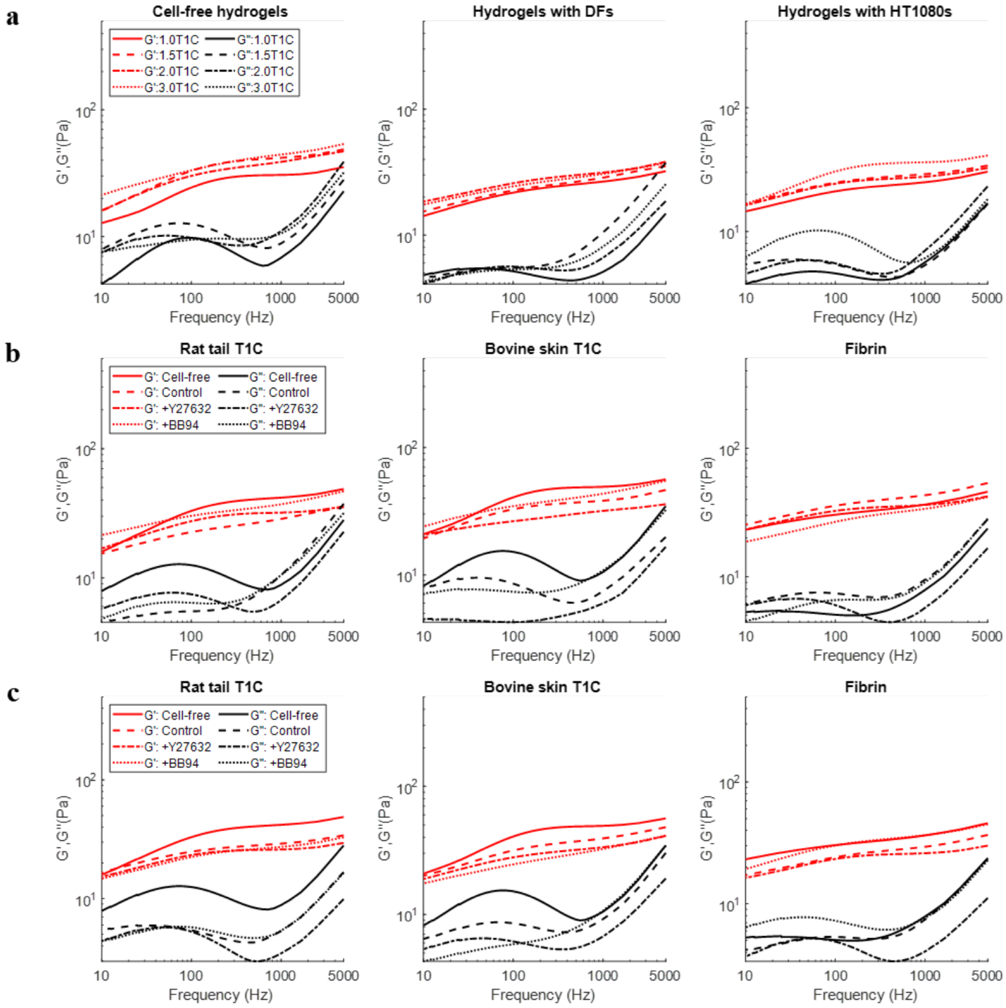

Figure S5. *G'* and *G"* values calculated from passive microrheology data (detection beam only). Change in *G'* and *G"* with frequency inside (a) rat tail T1C hydrogels polymerized at different concentrations and probed around (b) DFs and (c) HT1080s cultured in 3 different types of hydrogels (rat tail T1C, bovine skin T1C, fibrin), untreated or treated with Y27632 or BB94.

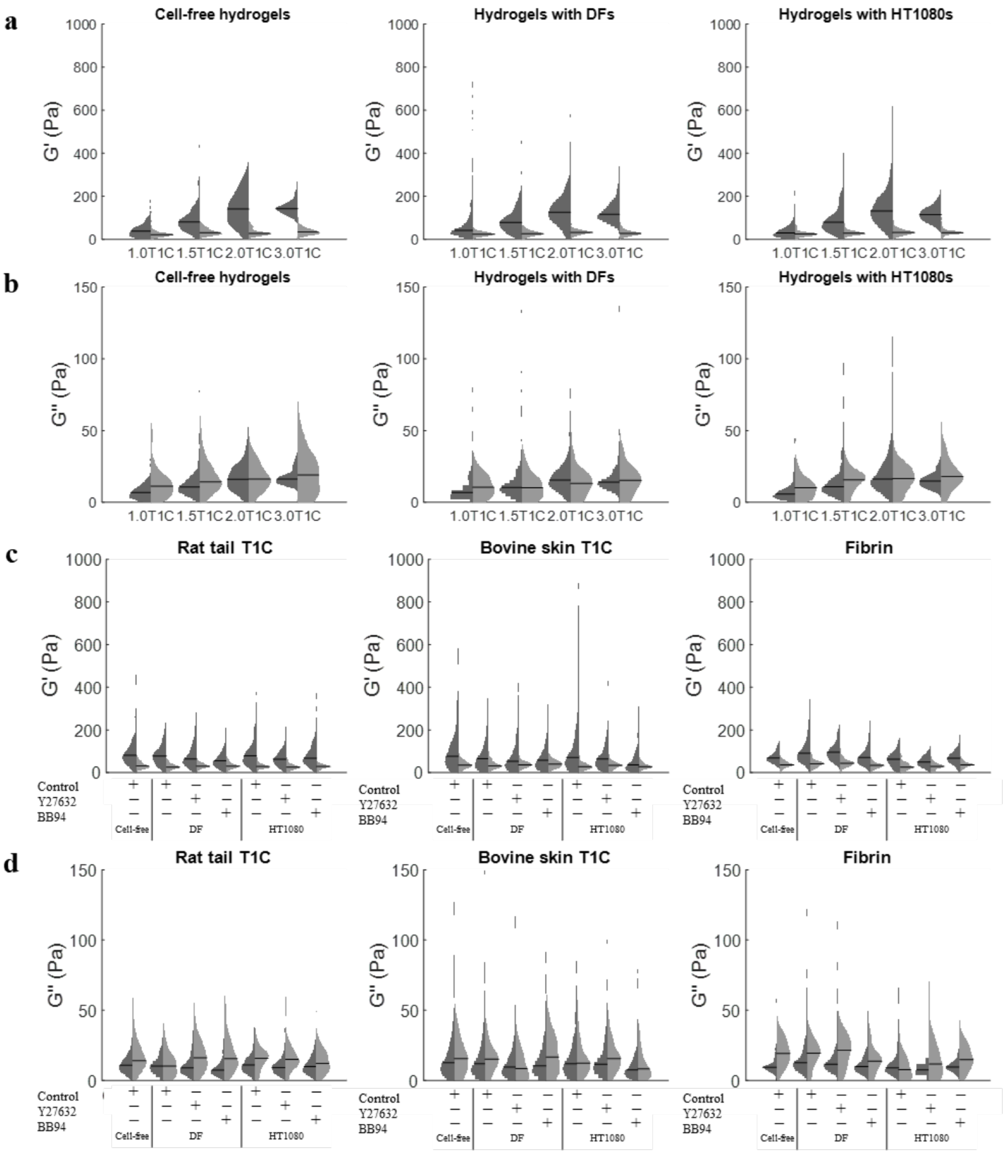

Figure S6. Comparison of *G'* and *G''* values measured using active (dark grey) and passive (light grey) microrheology. (a) *G'* and (b) *G''* distributions for rat tail T1C hydrogels polymerized at different concentrations. (c) *G'* and (d) *G''* distributions for 3 different types of hydrogels (rat tail T1C, bovine skin T1C, fibrin).

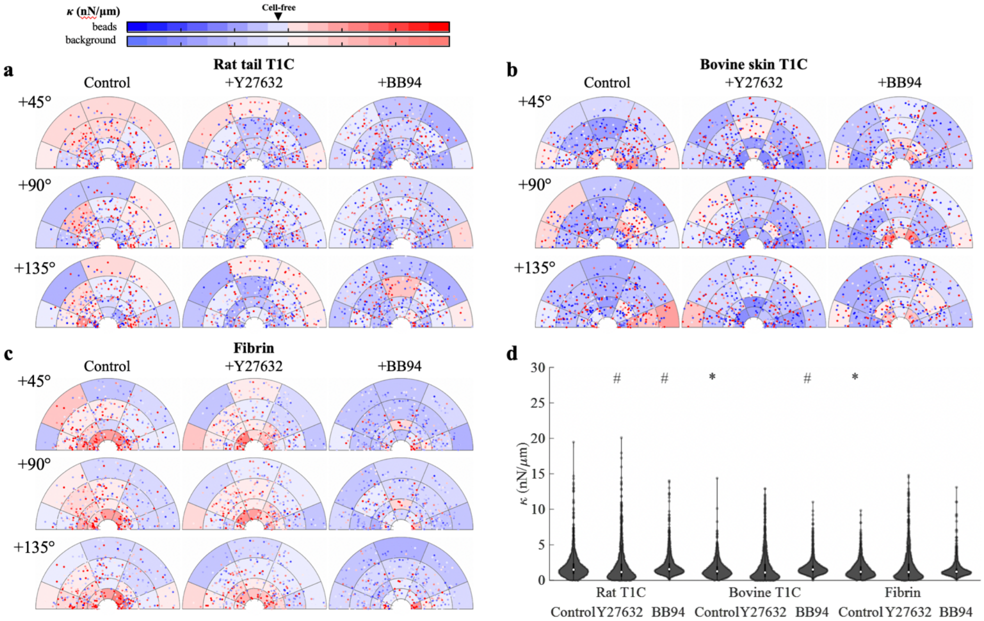

e

MER summary table - ECM comparison

log(κ) ~ distance + θ + oscillation angle + ECM			
Adjusted R^2 : 0.0536			
Coefficients:	Estimate	Std. Error	*p*
(intercept)	0.46	4.06e-2	< 0.01
distance	-1.69e-3	3.28e-4	< 0.01
θ	-1.71e-4	2.16e-4	0.43
oscillation angle (45°)	-1.85e-2	3.51e-2	0.60
oscillation angle (90°)	-4.95e-2	3.51e-2	0.16
oscillation angle (135°)	-2.16e-2	3.51e-2	0.54
ECM (bovine skin T1C)	-0.29	3.13e-2	< 0.01
ECM (fibrin)	0.19	3.06e-2	< 0.01

f

MER summary table - treatment comparison

log(κ) ~ distance + θ + oscillation angle + treatment

	**Rat tail T1C**			**Bovine skin T1C**			**Fibrin**		
	Adjusted R^2 : 0.0152			Adjusted R^2 : 0.0002			Adjusted R^2 : 0.1150		
Coefficients:	Estimate	Std. Error	*p*	Estimate	Std. Error	*p*	Estimate	Std. Error	*p*
(intercept)	0.33	3.55e-2	< 0.01	6.28e-2	5.74e-2	0.27	0.71	2.42e-2	< 0.01
distance	7.43e-4	2.82e-4	0.01	-3.98e-4	4.12e-4	0.33	-3.54e-3	1.82e-4	< 0.01
θ	-2.47e-4	1.94e-4	0.20	-1.18e-4	3.05e-4	0.70	3.31e-4	1.27e-4	0.01
oscillation angle (45°)	-1.13e-3	3.05e-2	0.97	-2.91e-2	4.83e-2	0.55	3.49e-3	2.03e-2	0.86
oscillation angle (90°)	-2.76e-2	3.05e-2	0.37	4.17e-4	4.83e-2	0.99	6.23e-3	2.03e-2	0.76
oscillation angle (135°)	-1.68e-2	3.05e-2	0.58	2.69e-2	4.83e-2	0.58	1.25e-3	2.03e-2	0.95
treatment (Y27632)	-0.18	2.71e-2	< 0.01	-6.86e-2	4.16e-2	0.10	-8.01e-2	1.74e-2	< 0.01
treatment (BB94)	-0.23	2.68e-2	< 0.01	2.99e-2	4.20e-2	0.48	-0.28	1.76e-2	< 0.01

Figure S7. Supplementary material to Figure 2d. Stiffness κ distribution around DFs probed at +45°, +90° and +135° with respect to the long axis of the cell. Data shown for DFs cultured in (a) rat tail T1C, (b) bovine skin T1C, and (c) fibrin. (d) Aggregated stiffness results in all directions. Statistical difference in κ assessed using Tukey-Kramer test across tested ECM types (as compared to rat tail T1C) or following Y27632 or BB94 (as compared to control condition) is denoted by * or #, respectively. MER summary table describing significant predictors of stiffness probed around DFs cultured in the 3 different types of hydrogels (e) and treated with Y27632 or BB94 (f). Discrete variables of oscillation axis, ECM and treatment were simply encoded with the reference to cell orientation angle, rat tail T1C and control condition, respectively.

Table S1. Supplementary material to (a) Figure 3b, (b) Figure 3c and (c) Figure 3d. Shown are the *p*-statistics using the Tukey-Kramer test.

a **Nuclear/Cytoplasmic YAP ratio**

		Rat tail T1C			Bovine skin T1C			Fibrin		
		Control	+Y27632	+BB94	Control	+Y27632	+BB94	Control	+Y27632	+BB94
Rat tail T1C	Control		0.23	0.95	> 0.99	0.98	> 0.99	> 0.99	> 0.99	> 0.99
	+Y27632			0.01	0.19	0.01	0.34	0.54	0.61	0.72
	+BB94				0.97	> 0.99	0.88	0.72	0.66	0.54
Bovine skin T1C	Control					0.99	> 0.99	> 0.99	> 0.99	> 0.99
	+Y27632						0.95	0.85	0.8	0.7
	+BB94							> 0.99	> 0.99	> 0.99
Fibrin	Control								> 0.99	> 0.99
	+Y27632									> 0.99
	+BB94									

b **% of fibronectin outside of the cell**

		Rat tail T1C			Bovine skin T1C			Fibrin		
		Control	+Y27632	+BB94	Control	+Y27632	+BB94	Control	+Y27632	+BB94
Rat tail T1C	Control		0.53	< 0.01	0.27	0.05	< 0.01	< 0.01	< 0.01	< 0.01
	+Y27632			< 0.01	> 0.99	0.97	0.19	< 0.01	< 0.01	< 0.01
	+BB94				< 0.01	0.01	0.47	0.02	0.25	0.98
Bovine skin T1C	Control					> 0.99	0.44	< 0.01	< 0.01	< 0.01
	+Y27632						0.87	< 0.01	< 0.01	< 0.01
	+BB94							< 0.01	< 0.01	0.03
Fibrin	Control								0.99	0.26
	+Y27632									0.87
	+BB94									

c **Solidity**

		Rat tail T1C			Bovine skin T1C			Fibrin		
		Control	+Y27632	+BB94	Control	+Y27632	+BB94	Control	+Y27632	+BB94
Rat tail T1C	Control		0.38	< 0.01	0.13	0.88	0.01	> 0.99	> 0.99	> 0.99
	+Y27632			0.77	> 0.99	0.01	0.87	0.57	0.68	0.14
	+BB94				0.97	< 0.01	> 0.99	0.01	0.02	< 0.01
Bovine skin T1C	Control					< 0.01	0.99	0.24	0.33	0.03
	+Y27632						< 0.01	0.73	0.63	0.99
	+BB94							0.02	0.03	< 0.01
Fibrin	Control								> 0.99	> 0.99
	+Y27632									0.99
	+BB94									

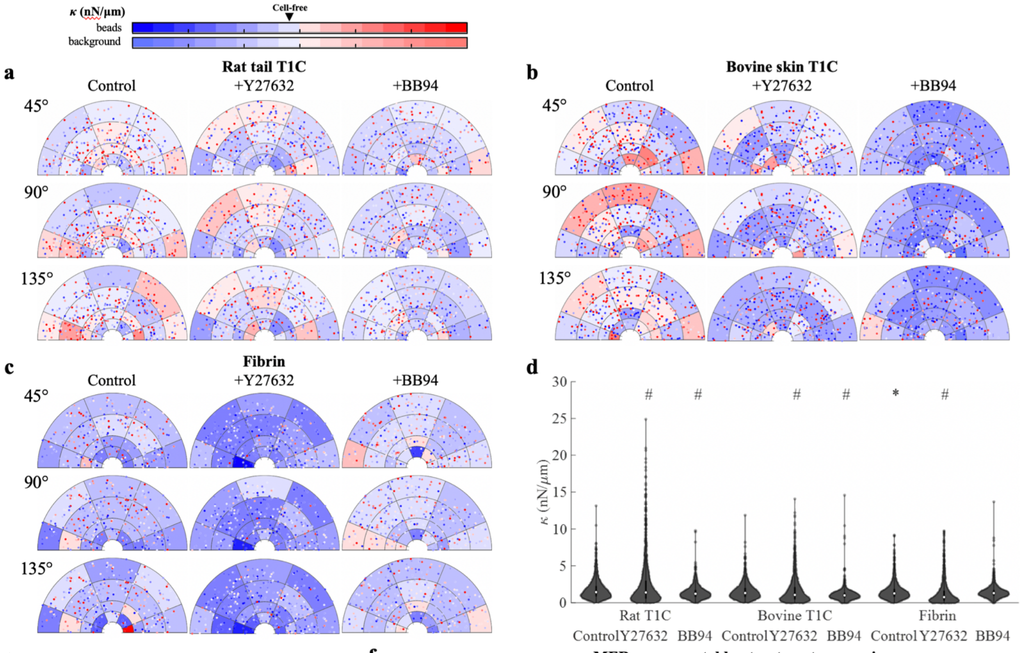

e **MER summary table - ECM comparison**

log(κ) ~ distance + θ + oscillation angle + ECM			
Adjusted R^2 : 0.0088			
Coefficients:	Estimate	Std. Error	*p*
(intercept)	0.26	4.57e-2	< 0.01
distance	7.85e-4	3.10e-4	0.01
θ	-3.34e-4	2.49e-4	0.18
oscillation angle (45°)	4.43e-2	3.70e-2	0.23
oscillation angle (90°)	2.28e-2	3.70e-2	0.54
oscillation angle (135°)	2.93e-2	3.70e-2	0.43
ECM (bovine skin T1C)	-8.68e-2	3.15e-2	0.01
ECM (fibrin)	-0.21	3.23e-2	< 0.01

f **MER summary table - treatment comparison**

log(κ) ~ distance + θ + oscillation angle + treatment

	**Rat tail T1C**			**Bovine skin T1C**			**Fibrin**		
	Adjusted R^2 : 0.0081			Adjusted R^2 : 0.0434			Adjusted R^2 : 0.0569		
Coefficients:	Estimate	Std. Error	*p*	Estimate	Std. Error	*p*	Estimate	Std. Error	*p*
(intercept)	0.24	3.50e-2	< 0.01	0.32	6.69e-2	< 0.01	7.67e-3	3.58e-2	0.83
distance	3.14e-4	2.22e-4	0.16	4.84e-4	4.21e-4	0.91	1.92e-3	2.34e-4	< 0.01
θ	3.52e-4	1.94e-4	0.07	-6.27e-4	3.53e-4	0.08	-6.66e-4	1.93e-4	< 0.01
oscillation angle (45°)	2.40e-2	2.81e-2	0.39	-3.20e-2	5.13e-2	0.53	1.34e-2	2.81e-2	0.63
oscillation angle (90°)	1.76e-2	2.81e-2	0.53	-6.22e-2	5.13e-2	0.23	-1.22e-2	2.81e-2	0.66
oscillation angle (135°)	2.18e-2	2.81e-2	0.44	-5.19e-2	5.13e-2	0.31	-3.29e-3	2.81e-2	0.91
treatment (Y27632)	-0.13	2.47e-2	< 0.01	-0.33	4.41e-2	< 0.01	-0.24	2.46e-2	< 0.01
treatment (BB94)	-0.15	2.44e-2	< 0.01	-0.64	4.47e-2	< 0.01	6.14e-2	2.47e-2	0.01

Figure S8. Supplementary material to Figure 4b. Stiffness κ distribution around HT1080s probed at 45°, 90° and 135° with respect to the horizontal axis of the image. Data shown for HT1080s cultured in (a) rat tail T1C, (b) bovine skin T1C, and (c) fibrin. (d) Aggregated stiffness results in all directions. Statistical difference in κ assessed using Tukey-Kramer test across tested ECM types (as compared to rat tail T1C) or following Y27632 or BB94 (as compared to control condition) is denoted by * or #, respectively. MER summary table describing significant predictors of stiffness probed around HT1080s cultured in the 3 different types of hydrogels (e) and treated with Y27632 or BB94 (f). Discrete variables of oscillation axis, ECM and treatment were simply encoded with the reference to horizontal axis of the image, rat tail T1C and control condition, respectively.

Table S2. Supplementary material to (a) Figure 5b, (b) Figure 5c and (c) Figure 5d. Shown are the *p*-statistics using the Tukey-Kramer test.

a — **Nuclear/Cytoplasmic YAP ratio**

		Rat tail T1C			Bovine skin T1C			Fibrin		
		Control	+Y27632	+BB94	Control	+Y27632	+BB94	Control	+Y27632	+BB94
Rat tail T1C	Control		< 0.01	0.04	> 0.99	> 0.99	0.32	0.94	< 0.01	> 0.99
	+Y27632			0.02	< 0.01	< 0.01	< 0.01	< 0.01	0.99	< 0.01
	+BB94				0.03	0.12	> 0.99	< 0.01	0.24	0.21
Bovine skin T1C	Control					> 0.99	0.27	0.95	< 0.01	> 0.99
	+Y27632						0.58	0.76	< 0.01	> 0.99
	+BB94							0.01	0.02	0.74
Fibrin	Control								< 0.01	0.61
	+Y27632									< 0.01
	+BB94									

b — **% of fibronectin outside of the cell**

		Rat tail T1C			Bovine skin T1C			Fibrin		
		Control	+Y27632	+BB94	Control	+Y27632	+BB94	Control	+Y27632	+BB94
Rat tail T1C	Control		0.01	0.84	0.06	> 0.99	0.79	< 0.01	< 0.01	< 0.01
	+Y27632			< 0.01	< 0.01	0.03	< 0.01	0.99	0.75	0.89
	+BB94				0.85	0.51	> 0.99	< 0.01	< 0.01	< 0.01
Bovine skin T1C	Control					0.01	0.93	< 0.01	< 0.01	< 0.01
	+Y27632						0.46	< 0.01	< 0.01	< 0.01
	+BB94							< 0.01	< 0.01	< 0.01
Fibrin	Control								> 0.99	> 0.99
	+Y27632									> 0.99
	+BB94									

c — **Circularity**

		Rat tail T1C			Bovine skin T1C			Fibrin		
		Control	+Y27632	+BB94	Control	+Y27632	+BB94	Control	+Y27632	+BB94
Rat tail T1C	Control		< 0.01	< 0.01	> 0.99	0.01	< 0.01	< 0.01	< 0.01	0.1
	+Y27632			0.07	< 0.01	0.04	0.15	< 0.01	0.08	< 0.01
	+BB94				0.01	> 0.99	> 0.99	< 0.01	> 0.99	< 0.01
Bovine skin T1C	Control					0.01	< 0.01	< 0.01	< 0.01	0.08
	+Y27632						> 0.99	< 0.01	> 0.99	< 0.01
	+BB94							< 0.01	> 0.99	< 0.01
Fibrin	Control								< 0.01	0.97
	+Y27632									< 0.01
	+BB94									
